# Characterization and Stress Response of the JmjC Domain-Containing Histone Demethylase Gene Family in the Allotetraploid Cotton Species *Gossypium hirsutum*

**DOI:** 10.3390/plants9111617

**Published:** 2020-11-20

**Authors:** Jie Zhang, Junping Feng, Wei Liu, Zhongying Ren, Junjie Zhao, Xiaoyu Pei, Yangai Liu, Daigang Yang, Xiongfeng Ma

**Affiliations:** 1 Zhengzhou Research Base, State Key Laboratory of Cotton Biology, School of Life Sciences, Zhengzhou University, Zhengzhou 450001, China; Zhangjie2935@163.com; 2 Collaborative Innovation Center of Henan Grain Crops, Agronomy College, Henan Agricultural University, Zhengzhou 450002, China; 18838916993@163.com; 3 State Key Laboratory of Cotton Biology, Institute of Cotton Research, Chinese Academy of Agricultural Sciences, Anyang 455000, China; renzhongying@caas.cn (Z.R.); zhaojunjie@caas.cn (J.Z.); peixiaoyu@caas.cn (X.P.); liuyangai@caas.cn (Y.L.); yangdaigang@caas.cn (D.Y.)

**Keywords:** *Gossypium*, polyploidization, histone methylation, *JmjC* genes, cold stress, osmotic stress

## Abstract

Histone modification is an important epigenetic modification that controls gene transcriptional regulation in eukaryotes. Histone methylation is accomplished by histone methyltransferase and can occur on two amino acid residues, arginine and lysine. JumonjiC (JmjC) domain-containing histone demethylase regulates gene transcription and chromatin structure by changing the methylation state of the lysine residue site and plays an important role in plant growth and development. In this study, we carried out genome-wide identification and comprehensive analysis of *JmjC* genes in the allotetraploid cotton species *Gossypium hirsutum*. In total, 50 *JmjC* genes were identified and in *G. hirsutum*, and 25 *JmjC* genes were identified in its two diploid progenitors, *G. arboreum* and *G. raimondii*, respectively. Phylogenetic analysis divided these *JmjC* genes into five subfamilies. A collinearity analysis of the two subgenomes of *G. hirsutum* and the genomes of *G. arboreum* and *G. raimondii* uncovered a one-to-one relationship between homologous genes of the *JmjC* gene family. Most homologs in the *JmjC* gene family between A and D subgenomes of *G. hirsutum* have similar exon-intron structures, which indicated that *JmjC* family genes were conserved after the polyploidization. All *G. hirsutum* *JmjC* genes were found to have a typical JmjC domain, and some genes also possess other special domains important for their function. Analysis of promoter regions revealed that *cis*-acting elements, such as those related to hormone and abiotic stress response, were enriched in *G. hirsutum JmjC* genes. According to a reverse transcription-quantitative polymerase chain reaction (RT-qPCR) analysis, most *G. hirsutum* *JmjC* genes had high abundance expression at developmental stages of fibers, suggesting that they might participate in cotton fiber development. In addition, some *G. hirsutum* *JmjC* genes were found to have different degrees of response to cold or osmotic stress, thus indicating their potential role in these types of abiotic stress response. Our results provide useful information for understanding the evolutionary history and biological function of *JmjC* genes in cotton.

## 1. Introduction

Epigenetics is the study of changes in gene expression that can be inherited without changes in the DNA sequence [1]. Gene expression is controlled through epigenetic regulation, which mainly includes DNA methylation, histone modification, chromatin remodeling and non-coding RNA regulation [2]. Eukaryotic genomic DNA is tightly packed into chromatin. Nucleosomes, which are the basic structural units of chromatin, consist of 146 bp of DNA wrapped around a histone octamer core composed of two molecules each of H2A, H2B, H3 and H4 [3]. Post-translational covalent modification of histones is an important mode of epigenetic regulation that regulates gene expression and other processes by affecting the state of chromatin [4]. The N-terminal amino acid tails of histones are often covalently modified, mainly through acetylation, methylation, ubiquitination, phosphorylation, glycosylation, ADP-ribosylation and SUMOylation [5]. Different covalent modifications occur at different sites of histones. For example, lysine can be acetylated, methylated and ubiquitinated, arginine can be methylated, and threonine and serine can be phosphorylated [6]. Histone methylation has important effects on gene transcription and chromatin structure that vary according to the location and degree of methylation. Histone methylation occurs mainly at the N-terminal arginine (R) and lysine (K) sites of histones H3 and H4, including K4, K9, K27, K36 of H3 and K20 for H4. Lysine residues can occur in three methylation forms, namely, monomethylated (Kme1), dimethylated (Kme2), or trimethylated (Kme3), whereas arginine can only undergo monomethylation (Rme1), symmetric dimethylation (Rme2s), or asymmetric dimethylation (Rme2a) [7]. Histone lysine methylation has several roles, not only affecting the static charge of modified residues, but also improving hydrophobicity and changing intramolecular or intermolecular interactions [5]. In general, methylation of histones H3K9 (H3K9me2/3) and H3K27 (H3K27me3) is associated with transcriptional inhibition, whereas methylation of H3K4 (H3K4me2/3) and H3K36 (H3K36me3) is associated with transcriptional activation [8]. Two histone lysine demethylases are currently known. The first, histone lysine-specific demethylase 1 (LSD1), reverses the monomethylation or dimethylation of lysine residues by flavin adenine dinucleotide (FAD), but does not act on trimethylated lysine [9]. Four proteins homologous to LSD1, namely, FLOWERING LOCUS D (FLD), LSD1-LIKE 1 (LDL1), LSD1-LIKE 2 (LDL2) and LSD1-LIKE 3 (LDL3), have been found in Arabidopsis and rice [10,11]. The second histone lysine demethylase, JumonjiC (JmjC) domain-containing histone demethylase, is functionally dependent on ferrous ion (Fe [II]) and α-ketoglutarate (α-KG) as prosthetic groups and is active on all three methylated lysines [12]. The first JmjC protein, which is essential for heart and brain development [13], was originally identified in mice and subsequently found to have demethylase activity [14]. More and more evidence suggested that the JmjC proteins constitute an important family of histone lysine demethylases and play an important role in maintaining stable histone methylation in vivo [9,15].

At present, the JmjC proteins in Arabidopsis are classified into five categories based on sequence similarity and catalytic specificity: KDM4/JHDM3 (*AtJMJ11-13*), KDM5/JARID (*AtJMJ14-19*), JMJD6 (*AtJMJ21/22*), KDM3/JHDM2 (*AtJMJ24-29*) and JmjC domain-only (*AtJMJ20* and *AtJMJ30-32*) subfamilies [12,16,17]. Members of different subfamilies act on different substrates. The KDM5/JARID subfamily can remove methylation from H3K4me1/2/3 [14]. The KDM4/JHDM3 subfamily can reverse the methylation of H3K9me2/3 and H3K36me2/3, while KDM3/JHDM2 subfamily proteins can demethylate H3K9me1 and H3K9me2 [18]. JMJD6 subfamily proteins have the activity of demethylation of H3K27me2/3 [12,19], and the JmjC domain-only subfamily can remove the methylation of H3K27me3 [20]. In Arabidopsis, the IBM1(*AtJMJ25*), belonging to the KDM3/JHDM2 subfamily, can remove the methylation modification of H3K9me1/2 within transcriptionally active genes and the CHG methylation of DNA [21,22]. ELF6 *(AtJMJ11)* and REF6 *(AtJMJ12*) in Arabidopsis belong to the KDM4/JHDM3 subfamily and have opposite functions in controlling flowering time. Deletion mutation of ELF6 (*AtJMJ11*) leads to early flowering [23,24]. REF6 *(AtJMJ12)* is a close homolog of ELF6 *(AtJMJ11)* and is the protein with H3K27me3 demethylase activity identified in plants, and deletion mutation of REF6 *(AtJMJ12)* results in delayed flowering time [25]. *AtJMJ14*, an active histone H3K4 demethylase, belongs to the KDM5/JARID subfamily; it can demethylate H3K4me1/2/3, negatively regulate the expressions of FLOWERING LOCUS T (FT) and SUPPRESSOR OF OVEREXPRESSION OF CONSTANS (SOC) genes, and control flowering time in Arabidopsis [26,27]. The KDM5/JARID subfamily also includes *AtJMJ15* and *AtJMJ18*, which can bind to FLOWERING LOCUS C (FLC) chromatin. Overexpression of *AtJMJ15* and *AtJMJ18* reduces H3K4 methylation levels in FLC chromatin and inhibits FLC expression, thereby promoting the expression of FT in accompanying cells to stimulate flowering, which leads to a significantly early flowering phenotype [28,29]. *AtJMJ30*, a member of the JmjC domain-only subfamily, is a participant in circadian rhythm regulation, mainly through its involvement in the regulation of circadian cycle length [30].

Moreover, the *JmjC* genes also play crucial roles in plant response to stresses. The overexpressed plants of *AtJMJ15* showed greater salt tolerance compared with the wild-type Arabidopsis, while the functionally deficient mutant was more sensitive to salt stress [31]. *AtJMJ17* participates in Arabidopsis response to dehydration stress and abscisic acid (ABA), and mutants with an inactivated form of this gene show dehydration stress tolerance and ABA hypersensitivity during stomatal closure [32]. In *Medicago truncatula*, *MtJMJC5* undergoes cold specifically induced alternative splicing, and the cold-dependent alternative splicing could be reversed after ambient temperature returning to the normal [33]. In *Brassica rapa*, the transcripts of *JmjC* genes were induced under hot and cold treatments [34]. In maize, all 19 *JmjC* genes were responsive to heat stress treatment [35]. In rice, *OsJMJ704* epigenetically suppressed defense negative regulator genes [36], while *OsJMJ705* epigenetically activated defense positive regulator genes [37], to enhance resistance to pathogen attack.

*Gossypium* constitutes approximately 50 species, including 45 diploid and 5 tetraploid species, with sporophytic chromosome counts of 26 (2*n* = 2*x* = 26) and 52 (2*n* = 4*x* = 52), respectively. On the basis of cytological studies, eight different genomes of diploid *Gossypium* species have been distinguished (A–G and K), while the five tetraploid species are allotetraploids possessing both A and D diploid genomes [38]. Two of the diploids, the wild species *Gossypium raimondii* and the cultivated cotton *G. arboreum*, harbor D and A genomes, respectively [39,40]. Approximately 1.5 million years ago, diploid cotton (*G. arboreum*) originating from Africa and diploid cotton (*G. raimondii*) from South America gave rise via hybridization and genome doubling to five allotetraploid species, namely, *G. hirsutum*, *G. barbadense*, *G. tomentosum*, *G. darwinii* and *G. mustelinum* [38,39]. Among them, *G. hirsutum* was domesticated and is now the main cultivated cotton species [39,41]. More than 95% of commercially obtained cotton fiber is from *G. hirsutum*, which is characterized by its high productivity and strong environmental adaptability [42,43]. Moreover, because cotton includes a wide variety of species with rich genetic diversity, it has important applications in the exploration of the origin, genomic evolution and polyploidy of plants [44,45].

Cotton is an economically important crop as well as a model system to studying polyploidization [45,46]. As one of the most important fiber crops, the allotetraploid cotton species, *G. hirsutum*, is the source of much of the natural fiber used in the textile industry worldwide [47,48]. However, the role and function of JmjC domain-containing histone demethylase genes in cotton is poorly understood, so requires further investigation. In this study, consequently, we identified the *JmjC* genes at the genome-wide level in *G. hirsutum*, and examined their phylogenetic relationships, chromosomal locations, collinearity, gene and domain structures, *cis*-elements in the promoters and spatiotemporal expressions. Our results provide insights into the evolution and function of *JmjC* genes in *G. hirsutum*.

## 2. Results

### 2.1. Identification of G. hirsutum JmjC Genes

Using a combination of methods, we identified 50 *JmjC* genes in the allotetraploid cotton species *G. hirsutum*, more than twice the number found in Arabidopsis (21) or rice (20). To better understand the evolutionary history of *G. hirsutum JmjC* genes, we searched for *JmjC* genes in the other two diploid cotton species, *G. arboreum* and *G. raimondii*, using the same method. We identified 25 *G. arboreum* and 25 *G. raimondii JmjC* genes, exactly half the number found in *G. hirsutum*. The newly identified *JmjC* genes in *G. raimondii* were first named *GrJMJ1*-*25* according to their order on chromosomes. The *JmjC* genes in *G. arboreum* were named *GaJMJs* based on the homologs in *G. raimondii* with the same number as in *G. raimondii*. The *JmjC* genes in *G. hirsutum* were named *GhJMJs*, corresponding to the homologs in *G. raimondii* and *G. arboreum*, with suffixes D and A added to represent D and A subgenomes, respectively. Basic features of *G. hirsutum JmjC* genes, including chromosomal location, protein length, molecular weight (Mw), isoelectric point (pI), and predicted subcellular localization, are listed in Table S1. An analysis of physicochemical characteristics revealed that amino acid number, Mw, and theoretical pI vary widely among *JmjC* gene family members in *G. hirsutum*. According to the results of subcellular prediction, most *JmjC* gene family members are located in the nucleus, which is consistent with their histone demethylation function.

### 2.2. Phylogenetic Analysis of G. hirsutum JmjC Genes

**Figure 1:**
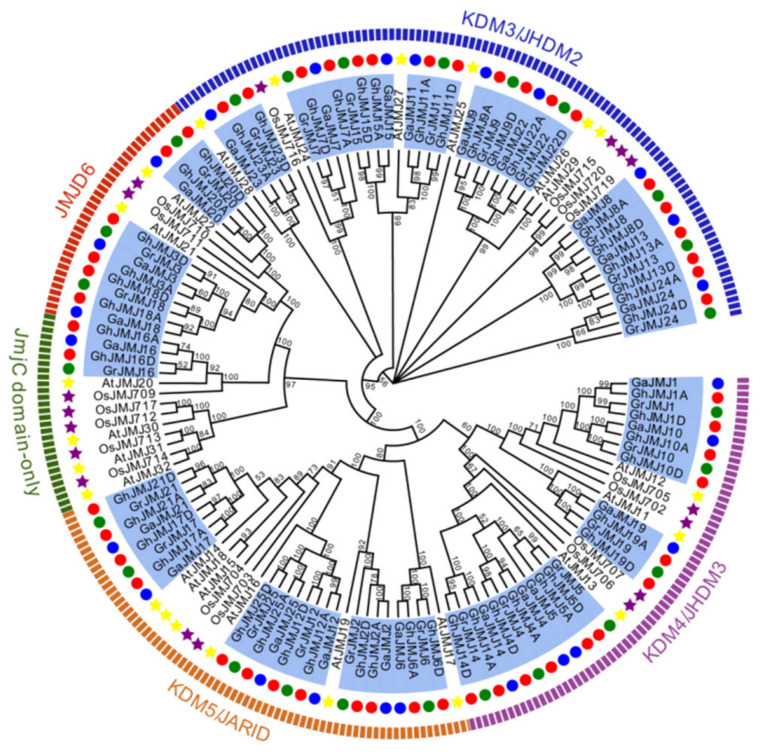
Phylogenetic tree of JmjC domain-containing histone demethylases from *G. hirsutum* (Gh), *G. arboreum* (Ga), *G. raimondii* (Gr), Arabidopsis (At) and rice (Os). Phylogenetic analysis was performed in MEGA 7.0 using the neighbor-joining method based on full-length protein sequences of *JmjC* genes from different plant species. Bootstrap support is indicated at respective nodes. Five clades are evident: KDM3/JHDM2, KDM4/JHDM3, KDM5/JARID, JMJD6 and JmjC domain-only. They are distinguished by blue, purple, orange, red and green arcs in turn. *G. hirsutum* (Gh), *G. arboreum* (Ga) and *G. raimondii* (Gr) *JmjC* genes are indicated by red, blue and green dots, respectively. Arabidopsis (At) and rice (Os) *JmjC* genes are indicated by yellow and purple stars, respectively.

To assess phylogenetic relationships of *JmjC* family genes, we performed multiple alignments of full-length protein sequences and JmjC domain sequences of *JmjC* genes from *G. hirsutum*, *G. arboreum*, *G. raimondii*, Arabidopsis and rice (Table S2). The evolutionary trees were constructed from the aligned sequences using MEGA 7.0 software, respectively. As shown in Figure 1 and Figure S1, the two phylogenetic trees presented very similar topologies, and JmjC domain-containing histone demethylases were separated into five groups: KDM3/JHDM2, KDM4/JHDM3, KDM5/JARID, JMJD6 and JmjC domain-only. The number of *G. hirsutum JmjC* genes differed among groups: 18 in the KDM3/JHDM2 subfamily, 12 each in KDM4/JHDM3 and KDM5/JARID, six in JMJD6, and two in JmjC domain-only.

### 2.3. Chromosomal Localization and Collinearity of G. hirsutum JmjC Genes

According to a chromosomal location analysis, *G. hirsutum JmjC* genes were distributed on 23 of the 26 chromosomes of *G. hirsutum* (Figure S2). The distribution of genes was as follows: four members on A12 and D12 chromosomes; three members each on chromosomes A05, A08, A10, D05, D08 and D10; two members each on A03, A07, A09, A11, A13, D07, D09, D11 and D13; and one member each on A01, A04, D01, D02, D03 and D04.

**Figure 2:**
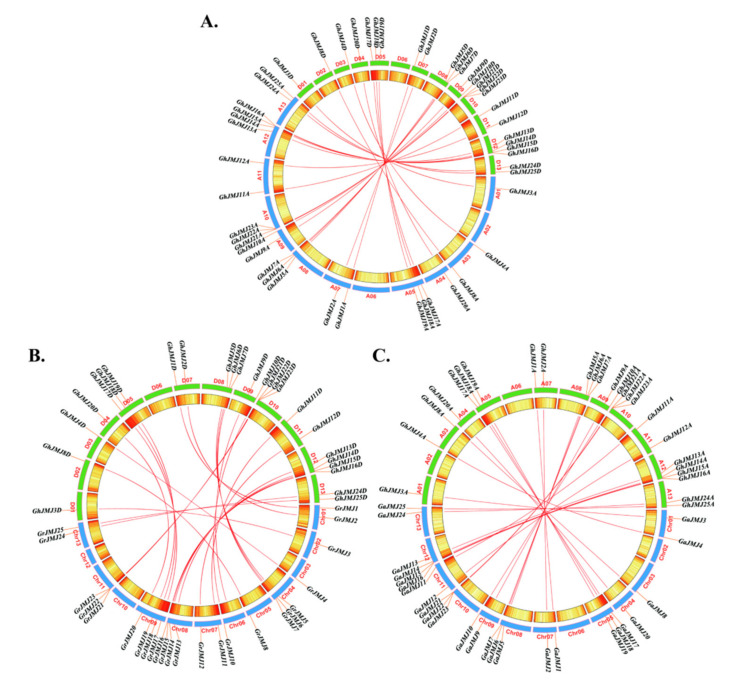
Locations and homologous relationships of *JmjC* genes in *G. raimondii*, *G. arboreum*, and A and D subgenomes of *G. hirsutum* (**A**–**C**). Locations and homologous relationships of *JmjC* family genes in the D and A subgenomes of *G. hirsutum* (**A**), the D subgenome of *G. hirsutum* and *G. raimondii* (**B**), and the A subgenome of *G. hirsutum* and *G. arboreum* (**C**). Chromosomes of *G. raimondii*, *G. arboreum*, and *G. hirsutum* D and A subgenomes are shown in different colors. The gene density of different regions is represented by a red and yellow gradient, where red indicates high gene density and yellow indicates low gene density. Putative homologs belonging to the *JmjC* gene family are connected by red lines.

*Gossypium hirsutum* is an allotetraploid cotton species with AA and DD genomes whose progenitors are widely believed to be the extant diploid cotton species *G. arboreum* and *G. raimondii* [40,43]. To explore the evolutionary history of the *JmjC* gene family in *Gossypium*, we performed synteny analysis to identify directly homologous *JmjC* genes in *G. hirsutum*, *G. arboreum* and *G. raimondii*. The locations of each *JmjC* gene and homologous gene pair uncovered in the analysis are shown in Figure 2. We identified 25 directly homologous gene pairs between the D subgenome of *G. hirsutum* and *G. raimondii*, 25 directly homologous gene pairs between the A subgenome of *G. hirsutum* and *G. arboreum*, and 25 directly homologous gene pairs between the D and A subgenomes of *G. hirsutum*. According to these results, a one-to-one relationship exists between homologs of the *JmjC* gene family in the two diploid cotton species and the two subgenomes of tetraploid cotton, which indicates that *JmjC* family genes have been highly conserved during cotton evolution.

### 2.4. Structural Features and Conserved Domains of G. hirsutum JmjC Genes

**Figure 3:**
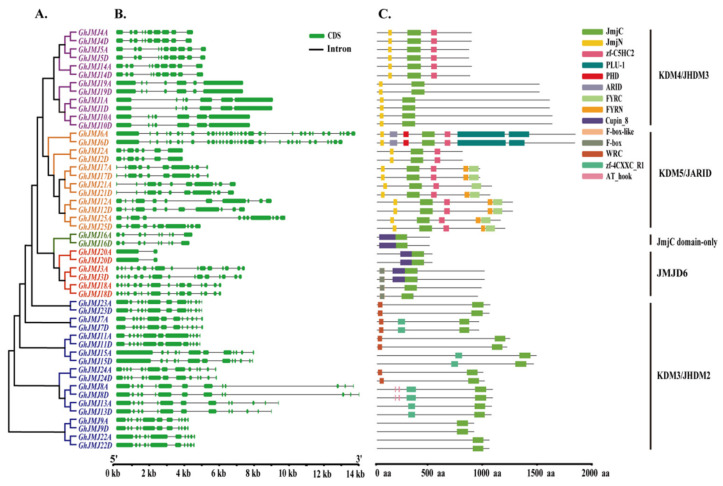
Phylogenetic relationships, gene structures and domain architectures of *G. hirsutum JmjC* genes. (**A**) Phylogenetic tree based on full-length protein sequences of *G. hirsutum JmjC* genes. Phylogenetic analysis was performed in MEGA 7.0 using the neighbor-joining method. Purple, orange, green, red and blue branches represent the KDM4/JHDM3, KDM5/JARID, JmjC domain-only, JMJD6 and KDM3/JHDM2 subfamilies, respectively. (**B**) Exon-intron structures of *G. hirsutum JmjC* genes. Black lines symbolize introns, and green boxes represent exons. The sizes of exons and introns can be estimated using the scale at the bottom. (**C**) The domain architectures of full-length JmjC domain-containing proteins. JmjC, Jumonji C domain; JmjN, Jumonji N domain; zf-C5HC2, C5HC2-type zinc finger; PLU-1, PLU-1 domain; PHD, plant homeobox domain; ARID, AT-rich interaction domain; FYRC, “FY-rich” domain C-terminal; FYRN, “FY-rich” domain N-terminal; Cupin_8, Cupin-like domain; F-box-like, F-box-like domain; F-box, F-box domain; WRC, Trp, Arg and Cys domain; zf-4CXXC_R1, Zinc-finger domain of monoamine-oxidase A repressor R1; AT_hook, DNA binding domain with preference for A/T rich regions.

Exon-intron structures play a crucial role in the evolution of organisms [49]. To understand the structural diversity of the *JmjC* gene family in *G. hirsutum*, we further analyzed exon-intron structures of these genes using the online Gene Structure Display Server v2.0 (Figure 3A,B). The number of exons in *G. hirsutum JmjC* genes varied from 2 to 33. Specifically, all genes possessed 7 to 16 exons except for *GhJMJ20A/GhJMJ20D* with 2 and *GhJMJ6A/GhJMJ6D* with 33. Most homologs in the *JmjC* gene family between A and D subgenomes of *G. hirsutum* have similar gene structures. For example, such gene pairs have highly consistent gene structures, exons/introns that are consistent in arrangement and number, and exons whose lengths are very similar. Exceptions exist, however, such as four gene pairs (*GhJMJ11A/GhJMJ11D*, *GhJMJ15A/GhJMJ15D*, *GhJMJ18A/GhJMJ18D* and *GhJMJ25A/GhJMJ25D*) have different intron numbers.

Conserved domains refer to domains that are invariant or identical over biological evolution or within a protein family and that generally have important functions that cannot be changed [50]. In histone demethylases containing the highly conserved JmjC domain, the activity of these proteins can be predicted by focusing on the conserved Fe (II) and α-KG binding sites within the domain [16]. To gain a better understanding of domain diversity, we determined the domain architectures of 50 *G. hirsutum* JmjC proteins by comparison with the full-length protein sequences of *JmjC* genes in the Pfam and SMART databases. As shown in Figure 3A,C all members of the *G. hirsutum JmjC* gene family were found to have a conserved JmjC domain, which is associated in JmjC proteins with demethylation of the histone lysine site [15]. The second most widely distributed domain was the JmjN domain, which appeared in all members of groups KDM4/JHDM3 and KDM5/JARID. All members of the KDM5/JARID subfamily also contained a zf-C5HC2 domain, which in combination with genes downstream of REF6 (*AtJMJ12*) allows the latter to assume the role of a histone demethylase [51]. The zf-C5HC2 domain was not observed in some members of subfamilies KDM4/JHDM3, possibly because the zf-C5HC2 domain in these proteins was less conserved and difficult to detect under the analysis parameters of this study. In addition to *GhJMJ2A/GhJMJ2D* and *GhJMJ6A/GhJMJ6D*, other members of the KDM5/JARID subfamily possess the FYRC domain that may have chromatin binding activity or contribute to the function of the JmjC domain by interacting with other proteins [16]. It is also found that *GhJMJ6A* and *GhJMJ6D* have three unique structural domains, the ARID domain, PHD domain and PLU-1 domain. The ARID domain is related to sequence-specific DNA binding [52], the PHD domain specifically recognize methylated (modified) histone codes and may be important readers of histone codes [53], and the PLU-1 domain plays a critical role in gene regulation and chromatin stability [54]. In the JMJD6 subfamily, four *JmjC* genes (*GhJMJ3A/GhJMJ3D* and *GhJMJ18A/GhJMJ18D*) containing the F-box domain are consistent with previous study [55].

### 2.5. Cis-Elements in the Promoter Regions of G. hirsutum JmjC Genes

**Figure 4:**
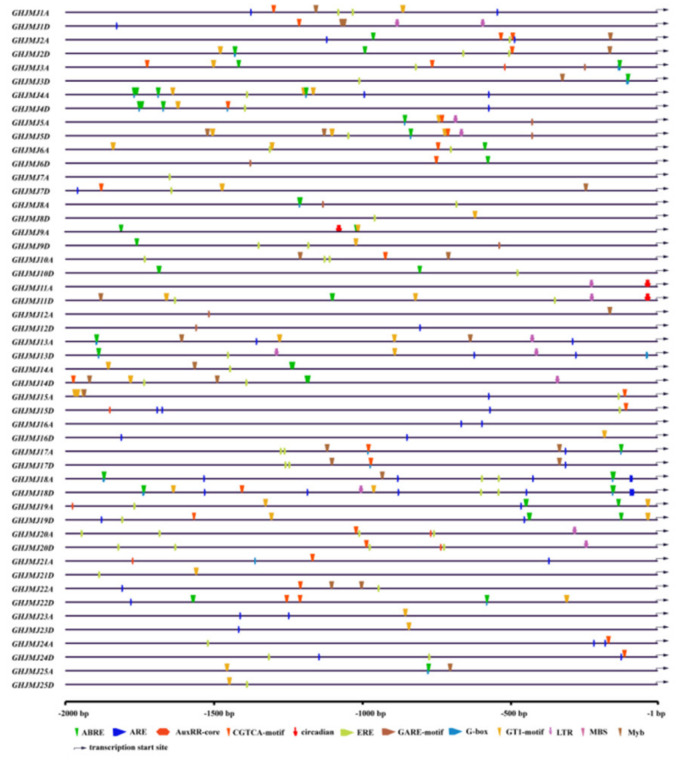
Distribution of *cis*-elements associated with major stresses in promoter sequences of *G. hirsutum JmjC* genes. Putative ABRE, ARE, AuxRR-core, CGTCA-motif, circadian, ERE, GARE-motif, G-box, GT1-motif, LTR, MBS, Myb and core sequences are represented by different symbols, as indicated at the bottom of the figure. ABRE, *cis*-acting element involved in abscisic acid responsiveness; ARE, anaerobic-induced essential regulatory element; AUXRR-core, an auxin-related element; CGTCA-motif, *cis*-acting regulatory element involved in MeJA responsiveness; circadian, involved in circadian *cis*-regulating elements; ERE, ethylene-responsive element; GARE-motif, gibberellin-responsive element; G-box, *cis*-acting element with light effect; GT1-motif, involved in salt-stress response elements; LTR, *cis*-acting element involved in hypothermic stress response; MBS, related to drought induction; Myb, *cis*-acting element involved in response to drought, high temperature and low temperature.

A promoter region is a DNA sequence located upstream of the 5′-end of a gene that activates RNA polymerase to facilitate accurate binding to template DNA and the specificity of transcription initiation [56]. To further elucidate the possible regulatory mechanisms of *G. hirsutum JmjC* genes in abiotic stress response, we searched the PlantCARE database for *cis*-regulatory elements in *JmjC* promoter regions (Figure 4).

We detected 12 types of *cis*-regulatory elements related to hormones and stresses in the promoters of *G. hirsutum JmjC* genes. Hormone-related elements included ABREs (abscisic acid responsiveness), EREs (ethylene responsive element), CGTCA motifs (MeJA-responsiveness), AUXRR-core motifs (auxin-responsive element) and GAREs (gibberellin-responsive element). Plant hormones can affect plant growth and induce physiological responses to environmental stimuli [57,58]. ABREs, which are *cis*-acting elements involved in abscisic acid reactivity, promote stomatal closure to control water loss in dry conditions [48,59]. The GARE motif is a responsive element for gibberellin, which plays an important role during seed, reproductive and maturation stages [60]. The CGTCA motif, related to jasmonic acid, regulates plant growth and is involved in the development of germination, rooting, flowering, fruiting and senescence. This element also actively participates in mechanisms underlying defense against biotic and abiotic stress [61]. Other *cis*-acting promoter elements including LTR (low-temperature responsiveness), MBS (myeloblastosis binding sites) and Myb (v-myb avian myeloblastosis viral oncogene homolog) are pressure-dependent. For example, the Myb transcription factor family can improve plant resistance drought stress [62]. We also detected *cis*-acting elements known to respond to biological or abiotic stresses including G-box and GT1-motif (termed the glycosyltransferase 1 motif), circadian (*cis*-regulated elements involved in circadian rhythm) and AREs (anaerobic-induced essential regulated elements) in promoter regions of *G. hirsutum JmjC* genes.

### 2.6. Expression Profiling of G. hirsutum JmjC Genes in Different Tissues and under Abiotic Stresses

**Figure 5:**
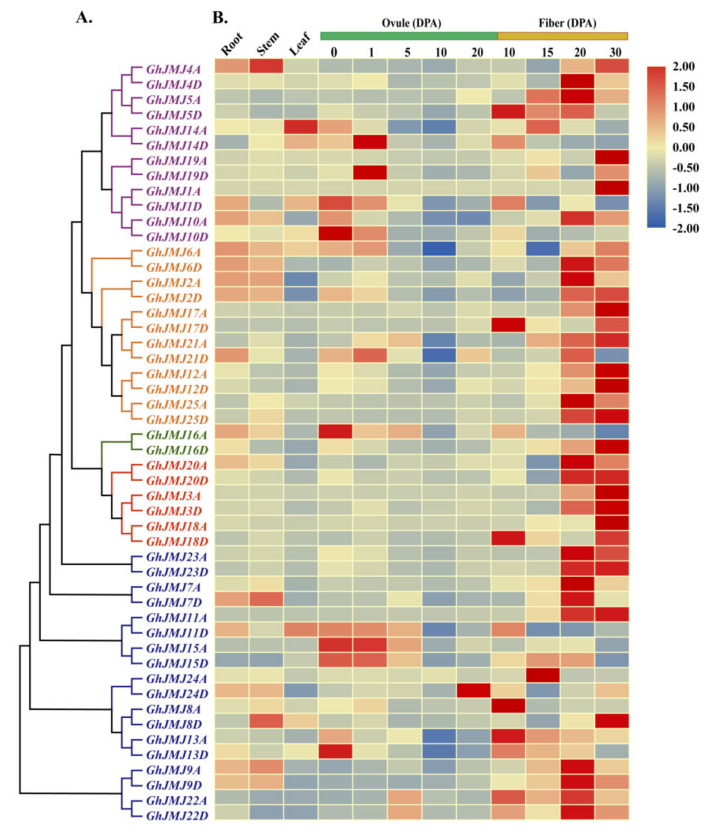
Heat map of expression profiles of *G. hirsutum JmjC* genes in different tissues. Expression levels were verified by reverse transcription-quantitative polymerase chain reaction (RT-qPCR). (**A**) Phylogenetic tree based on full-length protein sequences of *G. hirsutum JmjC* genes. Phylogenetic analysis was performed in MEGA 7.0 using the neighbor-joining method. Purple, orange, green, red and blue branches represent the KDM4/JHDM3, KDM5/JARID, JmjC domain-only, JMJD6 and KDM3/JHDM2 subfamilies, respectively. (**B**) Relative expression levels are indicated according to the color scale on the right, where blue and red respectively represent low and high transcript abundance. The type of tissue and the stage of development of ovules (green) and fibers (yellow) are indicated at the top of each column. DPA, days after anthesis.

To explore *JmjC* gene expression patterns during cotton development, we used reverse transcription-quantitative polymerase chain reaction (RT-qPCR) to measure *JmjC* gene expressions in roots, stems, leaves, ovules (0, 1, 5, 10 and 20 DPA) and fibers (10, 15, 20 and 30 DPA) (Figure 5A,B and Figure S3). As shown in the heat map revealing the expression patterns of different *G. hirsutum JmjC* genes, the majority of the homologous genes in the *JmjC* family have similar expression patterns. Most *JmjC* family members had their highest expression in 20 and 30 DPA fibers. Some family members had higher expression levels in 10 and 15 DPA fibers (*GhJMJ5D, GhJMJ8A, GhJMJ13A, GhJMJ17D, GhJMJ18D* and *GhJMJ24A*). A few genes were also highly expressed in 0 and 1 DPA ovules (*GhJMJ1D, GhJMJ10D, GhJMJ11D, GhJMJ13D, GhJMJ14D, GhJMJ15A, GhJMJ15D* and *GhJMJ19D*). In contrast, expression levels in root, stem and leaf tissues were generally low. According to the RT-qPCR results, *G. hirsutum JmjC* genes may play a role in the development of *G. hirsutum* fibers.

**Figure 6:**
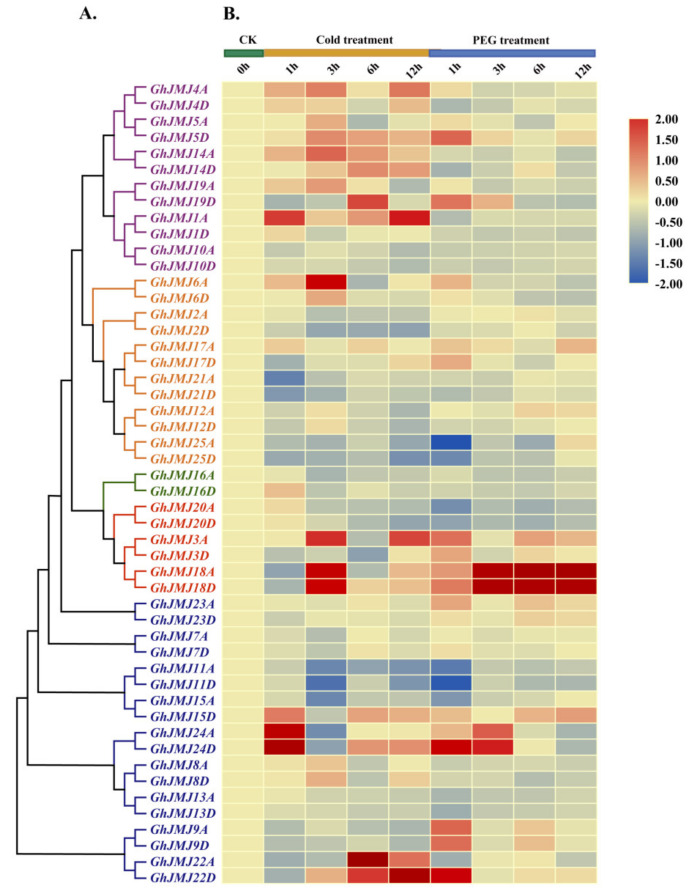
Heat map of expression profiles of *G. hirsutum JmjC* genes under cold and polyethylene glycol (PEG) treatments. Expression levels were verified by RT-qPCR. (**A**) Phylogenetic tree based on full-length protein sequences of *G. hirsutum JmjC* genes. Phylogenetic analysis was performed in MEGA 7.0 using the neighbor-joining method. Purple, orange, green, red and blue branches represent the KDM4/JHDM3, KDM5/JARID, JmjC domain-only, JMJD6 and KDM3/JHDM2 subfamilies, respectively. (**B**) Relative expression levels are indicated according to the color scale on the right, where blue and red respectively represent low and high transcript abundance. The type and duration of treatment (CK, cold stress, or osmotic stress) are indicated at the top of the map.

To further confirm the response of *G. hirsutum JmjC* genes to abiotic stress, we carried out a RT-qPCR analysis on all genes in the *G. hirsutum JmjC* family using cotton subjected to cold and polyethylene glycol (PEG) treatments (Figure 6A,B; Figures S4 and S5). According to the expression profiling, all genes were differentially expressed in leaves in response to cold stress (4 °C) or osmotic stress (PEG) compared with normal conditions (0 h). Most members of the *G. hirsutum JmjC* gene family were up-regulated in response to cold stress or osmotic stress. *GhJMJ1A, GhJMJ3A, GhJMJ4A, GhJMJ6A, GhJMJ14A, GhJMJ14D, GhJMJ18A, GhJMJ18D, GhJMJ19D, GhJMJ22A, GhJMJ22D, GhJMJ24A* and *GhJMJ24D* were significantly up-regulated under cold stress compared with non-treated controls. Under osmotic stress conditions of different durations, *GhJMJ3A, GhJMJ5D, GhJMJ9A, GhJMJ9D, GhJMJ18A, GhJMJ18D, GhJMJ19D, GhJMJ22D, GhJMJ24A* and *GhJMJ24D* were obviously up-regulated. Seven *G. hirsutum JmjC* genes (*GhJMJ3A, GhJMJ18A, GhJMJ18D, GhJMJ19D, GhJMJ22D, GhJMJ24A* and *GhJMJ24D*) were thus obviously up-regulated both under cold and osmotic stress conditions. These results suggest that these *G. hirsutum JmjC* genes respond to cold and osmotic stresses and have potential roles during cold and drought conditions.

## 3. Discussion

In plants, histone methylation plays a crucial role in a variety of biological processes, ranging from transcriptional regulation to heterochromatin formation. Different levels of methylation are responsible for different biological functions and are, therefore, essential for plant growth and development [5]. Histone demethylases containing the JmjC domain, which is highly conserved in plants, exert a key role in maintaining homeostasis of histone methylation *in vivo* [5,63]. Proteins containing the JmjC domain constitute an important family of histone lysine demethylases in animals and plants and play an important role in histone modification, which is an essential aspect of epigenetics [64]. At the genome-wide level, the *JmjC* gene family has been analyzed to account for its evolutionary history and biological function in certain plant species. For example, the evolution of histone demethylase has been studied in some green plant lineages [65], and 21 members in Arabidopsis [66], 48 members in soybean [67], 22 members in woodland strawberry [68], 28 members in *Rosa chinensis* [69], 20 members in rice [70] and 19 members in maize [35] have been identified and analyzed for the *JmjC* gene family, respectively. In contrast, no systematic study has been carried out on the *JmjC* gene family in *G. hirsutum*. In the present study, we conducted a comprehensive analysis of *G. hirsutum JmjC* genes, including their phylogenetic relationships, gene structures, domain structures, chromosomal locations, collinearity, promoter elements and expression profiles.

In this study, we identified 50 *JmjC* genes in the *G. hirsutum* genome for the first time and also detected 25 genes each in *G. arboreum* and *G. raimondii*, which is higher than the number found in Arabidopsis (21) and rice (20). Phylogenetic analysis of *JmjC* genes from five plant species divided cotton *JmjC* genes into five main subclasses. The KDM3/JHDM2 subfamily was found to contain the largest number of genes, accounting for 36% of the total. We also analyzed the structure of *G. hirsutum* JmjC proteins and thereby uncovered some preliminary details of their structural characteristics. Members of the KDM5/JARID subfamily, except for *GhJMJ2A, GhJMJ2D*, *GhJMJ6A GhJMJ6D* and *GhJMJ21A*, have the same domain, and they also share FYRC domains. *AtJMJ14* has been shown to play a role in Arabidopsis flowering time control through the interaction of FYRC domains with transcription factors [27]. The *G. hirsutum JmjC* genes *GhJMJ6A* and *GhJMJ6D* in the KDM5/JARID subfamily also contain three domains (ARID, PHD and PLU-1) that are related to DNA binding and gene regulation [53,54,71]. In Arabidopsis the homologous gene of *GhJMJ6A* and *GhJMJ6D* is *AtJMJ17*, which binds directly to the chromatin of OPEN STOMATA 1 (OST1) and demethylated H3K4me3 to regulate OST1 mRNA abundance, thereby regulating response to dehydration stress [32]. *GhJMJ6A* and *GhJMJ6D* may, therefore, have similar functions in cotton.

*Gossypium hirsutum*, as an AADD allotetraploid species, evolved from hybridization and genome doubling of A genome diploid species (*G. arboreum*) and D genome diploid species (*G. raimondii*) [40,43]. Through analysis of the locations and homologous relationships of *JmjC* genes in *G. raimondii*, *G. arboreum*, and A and D subgenomes of *G. hirsutum*, the 50 *JmjC* genes in *G. hirsutum* A and D subgenomes have a one-to-one direct homology with the 25 *JmjC* genes located in *G. raimondii* and *G. arboreum*, which indicates that these 50 genes are derived from the common ancestor of *G. raimondii* and *G. arboreum.* The number of *JmjC* genes in *G. hirsutum* is twice that of *G. raimondii* and *G. arboreum,* which indicates that all *JmjC* genes were retained during the process of polyploidization. Studies have shown that the *JmjC* gene family has diverse functions, including involvement in growth and development pathways, such as those related to flowering, photoperiod, hormone, and cell division and differentiation, and can reverse methylation at multiple sites, such as H3K4, H3K9, and H3K27. Moreover, some JmjC demethylases can remove methylation from multiple different sites [12]. The expansion of the number of *JmjC* genes may thus be related to the special characteristics of cotton or its adaptation to the environment.

To further study the possible functions of *JmjC* genes during *G. hirsutum* growth and development, we used RT-qPCR to reveal the spatio-temporal transcription patterns of these genes in 12 tissues at different stages of *G. hirsutum* development. Cotton fibers are the longest single cells found in plants. Many studies have shown that the morphological development of cotton fibers takes place in four stages: fiber initiation (on or near the day of anthesis), cell elongation (from the day of anthesis to approximately 21 to 26 DPA), secondary wall deposition and maturation (At approximately 45 to 60 DPA, the seed capsule dehisces and the thin fiber cells quickly dehydrate). Each of these stages is critical to fiber development [72]. According to the RT-qPCR results, most *G. hirsutum JmjC* genes clearly had similar expression patterns, that is, higher expression levels during fiber development occurring at 10–30 DPA, especially 20 and 30 DPA. In Arabidopsis, mutations of the REF6 *(AtJMJ12)* gene cause impaired cell elongation, and REF6 *(AtJMJ12)* and ELF6 *(AtJMJ11)* can also interact with the transcription factor BRI1 EMS SUPPRESSOR 1 (BES1) in the brassinosteroid (BR) signaling pathway to regulate the expression of BR reaction genes [24]. In addition, REF6 *(AtJMJ12)* promotes lateral root formation by eliminating the inhibition of PIN1/3/7 genes [73]. The homologs of REF6 *(AtJMJ12)* and ELF6 *(AtJMJ11)* in *G. hirsutum*, *GhJMJ10A* and *GhJMJ19A*, are highly expressed in 20 and 30 DPA fibers, which suggests that these *G. hirsutum JmjC* genes are involved in cotton fiber development.

*Cis*-acting elements in gene promoter regions play an important role in gene transcription initiation and participate in the regulation of gene expression [56]. We analyzed promoter sequences of *G. hirsutum JmjC* genes and uncovered important *cis*-acting elements, such as those related to hormone and abiotic stress responses. We thus speculated that the *G. hirsutum JmjC* family genes have functions in plant response to abiotic stress. To confirm this hypothesis, we used RT-qPCR to analyze expression patterns of *JmjC* genes in *G. hirsutum* plants subjected to cold and osmotic treatments. In Arabidopsis, *AtJMJ17* affects the expression of such genes by regulating the H3K4me3 level of the stress response genes encoding stomatal opening factor (OST1) and ABA INSENSITIVE 5 (ABI5) and plays a negative regulatory role in osmotic stress and ABA response [32]. According to the expression analysis in this study, the *AtJMJ17* gene homologs *GhJMJ6A/GhJMJ6D* were highly expressed under both cold and osmotic stresses. At the same time, the *cis*-acting element analysis shows that *GhJMJ6A/GhJMJ6D* also contains ABRE *cis*-acting element. We thus speculate that these genes are closely related to the response of *G. hirsutum* to cold or osmotic stress. Our RT-qPCR analysis of these differentially expressed genes has thus provided novel insights into plant responses to cold and osmotic stresses.

## 4. Materials and Methods

### 4.1. Identification of JmjC Genes in G. hirsutum and other Cotton Species

*Gossypium hirsutum*, *G. arboreum* and *G. raimondii* genomic data were downloaded from the CottonFGD database [74] and used to create a local BLAST database. To identify *JmjC* genes in cotton, BLASTP and TBLASTN searches were carried out using the 21 Arabidopsis JmjC protein sequences [66] as queries. All identified candidate genes were analyzed online using Pfam [75] and SMART [76] databases to check for JmjC domains (PF02373).

Physicochemical parameters of *G. hirsutum JmjC* genes, including molecular weight (Mw) and isoelectric point (pI), were calculated using the compute pI/Mw tool in EXPASy [77], with the parameter set to average. Subcellular localization predictions of *G. hirsutum JmjC* proteins were carried out online using CELLO v2.5 [78].

### 4.2. Phylogenetic Analysis of G. hirsutum JmjC Genes

The amino acid sequences were aligned using the ClustalW program [79] with default parameters. According to previous published studies [35,67,69], the phylogenetic tree was then constructed using the neighbor-joining method as implemented in MEGA 7.0 [80] with the pairwise deletion option. Bootstrapping based on the p-distance model and 1000 replicates was conducted to assess internal branch reliability.

### 4.3. Chromosomal Mapping and Synteny Analysis of G. hirsutum JmjC Genes

The physical location data of *JmjC* genes were retrieved from the *Gossypium* genomic databases. The MapInspect software was used to generate chromosome distribution images [81]. The synteny analysis was performed as previously reported [82]. The chromosomal distribution and collinearity of *G. arboreum, G. raimondii* and *G. hirsutum JmjC* genes were then illustrated using the Advanced Circos circle diagram function in TBtools [83].

### 4.4. Structural Analysis of G. hirsutum JmjC Genes

To construct exon-intron structures that included the location and length of each *G. hirsutum JmjC* gene, coding sequences were compared with corresponding genomic sequences using the online Gene Structure Display Server v2.0 [84]. Conserved domains in JmjC domain-containing proteins encoded by *G. hirsutum JmjC* genes were identified through searches of Pfam [75] and SMART [76] databases with default parameters.

### 4.5. Prediction of Cis-Acting Elements in Promoter Regions of G. hirsutum JmjC Genes

To identify potential stress-response and hormone-related *cis*-acting elements in promoter sequences of *G. hirsutum JmjC* genes, the 2-kb sequence upstream of the initiation codon (ATG) of each *G. hirsutum JmjC* gene was compared against the PlantCARE database [85]. Adobe Illustrator software was used for the final figure construction.

### 4.6. Plant Materials and Stress Treatments

*Gossypium hirsutum* acc. TM-1 were used in the study. Roots, stems and leaves were collected from two-week-old seedlings, ovules were collected at 0, 1, 5, 10 and 20 DPA, and fibers were collected at 10, 15, 20 and 30 DPA. Cotton seedlings were planted in a liquid culture medium and grown until the third true leaf appeared in a plant growth chamber at 28 °C under a 16/8 h light/dark photoperiod. For the cold treatment, seedlings were incubated at 4 °C. For PEG treatment, seedlings were irrigated with 20% PEG 6000 solution. After cold and PEG treatments, the leaves were collected at 0, 1, 3, 6 and 12 h. All samples immediately were frozen in liquid nitrogen and stored at −80 °C.

### 4.7. Expression Analysis of G. hirsutum JmjC Genes

The RT-qPCR analysis was performed to reveal expression patterns of JmjC genes in representative tissues, namely, roots, stems, leaves, ovules (0, 1, 5, 10 and 20 DPA), and fibers (10, 15, 20 and 30 DPA). We also used the RT-qPCR analysis to examine expression patterns of JmjC genes in leaves of plants subjected to cold or PEG stress for 0, 1, 3, 6 and 12 h. Total RNA was extracted from different samples using an RN533-EASYspin Plus RNA rapid extraction kit (Adlai, Beijing, China). First-strand cDNA synthesis was carried out using 1 µg of total RNA and a PrimeScript first-strand cDNA synthesis kit (Takara, Dalian, China). Gene-specific primers (Table S3) were designed based on JmjC nucleotide sequences with Oligo software (v7.60, Molecular Biology Insights, Cascade, CO, USA). Reverse-transcription PCR was performed using the following protocol: 94 °C for 5 min; followed by 35 cycles of 94 °C for 30 s, 60 °C for 30 s, and 72 °C for 1 min; and the 72 °C for 10 min. The amplified fragment was purified with a FastPure Gel DNA Extraction mini kit (Vazyme, Nanjing, China) and cloned into a PMD18-T Vector (Takara, Dalian, China) for sequence verification. RT-qPCR was carried out on a RocheLightCycler480 instrument (Roche, Basel, Switzerland) the amplification parameters were as follows: stage (1): 95 °C, 5 min; stage (2): 40 cycles of 95 °C for 10 s, 60 °C for 10 s, 72 °C for 10 s; stage (3): extension at 72 °C for 10 min. The GhHIS3 gene was used as an internal control, with three biological replicates per sample. The 2-ΔCT was used to calculate the relative mRNA levels of each gene in different tissues. The 2−ΔΔCT was used to calculate the relative mRNA level of each gene in leaves under the cold and osmotic stress. A heatmap was generated using the HeatMap IIIustrator function in TBtools [83].

## 5. Conclusions

In this study, we conducted the first-ever comprehensive analysis of the *G. hirsutum JmjC* gene family. Using bioinformatics methods, we carried out a detailed investigation of phylogenetic relationships, chromosomal locations, collinear relationships, exon-intron structures, conserved domains and promoter elements of *G. hirsutum JmjC* family members. We also analyzed their expression patterns in different tissues and under cold and osmotic stresses.

The 50 *G. hirsutum JmjC* genes identified in this study were divided into five groups based on the result of phylogenetic analysis. The chromosomal localization showed that these *JmjC* genes are distributed at different densities on 23 of the 26 chromosomes of *G. hirsutum*. A collinearity analysis revealed that a one-to-one homologous relationship exists between *JmjC* genes in the two diploid cotton species *G. arboreum* and *G. raimondii* or in the two subgenomes of *G. hirsutum*. Most homologs in the *JmjC* gene family between A and D subgenomes of *G. hirsutum* have similar exon-intron structures, suggesting that the *JmjC* genes are strongly conserved in *G. hirsutum*. All *G. hirsutum JmjC* genes possess a typical JmjC domain, and other special domains were also found in some genes. According to an analysis of *cis*-acting promoter elements, *G. hirsutum JmjC* genes contain elements responsive to hormonal or abiotic stresses and might play an important role in plant growth and development. A RT-qPCR analysis demonstrated that *G. hirsutum JmjC* genes might have an important role in the development of fibers. This analysis also revealed differing responses of *G. hirsutum JmjC* genes to cold and osmotic stresses. Most family members exhibited significantly higher expression levels under cold or osmotic stress, which indicates that these genes are closely related to *G. hirsutum* response to cold or osmotic stress.

These results provide valuable clues for future identification of the specific functions of this gene family and the genetic diversity of cotton and other dicotyledonous plants.

## Supplementary Materials

The following are available online at https://www.mdpi.com/2223-7747/9/11/1617/s1, Table S1. Basic information on *JmjC* genes in *G. hirsutum*. Table S2. Protein sequences of *JmjC* genes from *G. hirsutum*, *G. raimondii*, *G. arboreum*, Arabidopsis and rice. Table S3. Primers used in this study. Figure S1. Phylogenetic tree of JmjC domain-containing histone demethylases from *G. hirsutum* (Gh), *G. arboreum* (Ga), *G. raimondii* (Gr), Arabidopsis (At) and rice (Os). Phylogenetic analysis was performed in MEGA 7.0 using the neighbor-joining method based on JmjC domain sequences of *JmjC* genes from different plant species. Figure S2. Physical locations of *JmjC* genes in the *G. hirsutum* genome. Figure S3. Expression profiles of *G. hirsutum JmjC* genes in different tissues. The error bars indicate the standard deviation estimated from three biological replicates. Figure S4. Expression profiles of *G. hirsutum JmjC* genes under cold treatments. The error bars indicate the standard deviation estimated from three biological replicates. Figure S5. Expression profiles of *G. hirsutum JmjC* genes under PEG treatments. The error bars indicate the standard deviation estimated from three biological replicates.

## References

[1] Egger G., Liang G., Aparicio A., Jones P.A. (2004). Epigenetics in human disease and prospects for epigenetic therapy. Nature.

[2] Cairns B.R. (2009). The logic of chromatin architecture and remodelling at promoters. Nature.

[3] Holliday R. (1987). DNA methylation and epigenetic defects in carcinogenesis. Mutat. Res..

[4] Jenuwein T., Allis C.D. (2001). Translating the histone code. Science.

[5] Kouzarides T. (2007). Chromatin modifications and their function. Cell.

[6] Bhaumik S.R., Smith E., Shilatifard A. (2007). Covalent modifications of histones during development and disease pathogenesis. Nat. Struct Mol. Biol..

[7] Liu C., Lu F., Cui X., Cao X. (2010). Histone methylation in higher plants. Annu. Rev. Plant Biol..

[8] Mosammaparast N., Shi Y. (2010). Reversal of histone methylation: Biochemical and molecular mechanisms of histone demethylases. Annu. Rev. Biochem..

[9] Shi Y., Lan F., Matson C., Mulligan P., Whetstine J.R., Cole P.A., Casero R.A., Shi Y. (2004). Histone demethylation mediated by the nuclear amine oxidase homolog LSD1. Cell.

[10] Jiang D., Yang W., He Y., Amasino R.M. (2007). Arabidopsis relatives of the human lysine-specific Demethylase1 repress the expression of FWA and FLOWERING LOCUS C and thus promote the floral transition. Plant Cell.

[11] Zhou D.-X., Hu Y. (2010). Regulatory function of histone modifications in controlling rice gene expression and plant growth. Rice.

[12] Klose R.J., Kallin E.M., Zhang Y. (2006). JmjC-domain-containing proteins and histone demethylation. Nat. Rev. Genet..

[13] Takeuchi T., Yamazaki Y., Katoh-Fukui Y., Tsuchiya R., Kondo S., Motoyama J., Higashinakagawa T. (1995). Gene trap capture of a novel mouse gene, jumonji, required for neural tube formation. Genes Dev..

[14] Tahiliani M., Mei P., Fang R., Leonor T., Rutenberg M., Shimizu F., Li J., Rao A., Shi Y. (2007). The histone H3K4 demethylase SMCX links REST target genes to X-linked mental retardation. Nature.

[15] Tsukada Y., Fang J., Erdjument-Bromage H., Warren M.E., Borchers C.H., Tempst P., Zhang Y. (2006). Histone demethylation by a family of JmjC domain-containing proteins. Nature.

[16] Lu F., Li G., Cui X., Liu C., Wang X.J., Cao X. (2008). Comparative analysis of JmjC domain-containing proteins reveals the potential histone demethylases in Arabidopsis and rice. J. Integr. Plant Biol..

[17] Luo M., Hung F.-Y., Yang S., Liu X., Wu K. (2014). Histone lysine demethylases and their functions in plants. Plant Mol. Biol..

[18] Yamane K., Toumazou C., Tsukada Y., Erdjument-Bromage H., Tempst P., Wong J., Zhang Y. (2006). JHDM2A, a JmjC-containing H3K9 demethylase, facilitates transcription activation by androgen receptor. Cell.

[19] Allis C.D., Berger S.L., Cote J., Dent S., Jenuwien T., Kouzarides T., Pillus L., Reinberg D., Shi Y., Shiekhattar R. (2007). New nomenclature for chromatin-modifying enzymes. Cell.

[20] Jones M.A., Morohashi K., Grotewold E., Harmer S.L. (2019). Arabidopsis JMJD5/JMJ30 acts Independently of LUX ARRHYTHMO within the plant circadian clock to enable temperature compensation. Front. Plant Sci..

[21] Miura A., Nakamura M., Inagaki S., Kobayashi A., Saze H., Kakutani T. (2009). An Arabidopsis jmjC domain protein protects transcribed genes from DNA methylation at CHG sites. Embo. J..

[22] Saze H., Shiraishi A., Miura A., Kakutani T. (2008). Control of genic DNA methylation by a jmjC domain-containing protein in Arabidopsis thaliana. Science.

[23] Noh B., Lee S.H., Kim H.J., Yi G., Shin E.A., Lee M., Jung K.J., Doyle M.R., Amasino R.M., Noh Y.S. (2004). Divergent roles of a pair of homologous jumonji/zinc-finger-class transcription factor proteins in the regulation of Arabidopsis flowering time. Plant Cell.

[24] Yu X., Li L., Li L., Guo M., Chory J., Yin Y. (2008). Modulation of brassinosteroid-regulated gene expression by Jumonji domain-containing proteins ELF6 and REF6 in Arabidopsis. Proc. Natl. Acad. Sci. USA.

[25] Lu F., Cui X., Zhang S., Jenuwein T., Cao X. (2011). Arabidopsis REF6 is a histone H3 lysine 27 demethylase. Nat. Genet..

[26] Lu F., Cui X., Zhang S., Liu C., Cao X. (2010). JMJ14 is an H3K4 demethylase regulating flowering time in Arabidopsis. Cell Res..

[27] Ning Y.Q., Ma Z.Y., Huang H.W., Mo H., Zhao T.T., Li L., Cai T., Chen S., Ma L., He X.J. (2015). Two novel NAC transcription factors regulate gene expression and flowering time by associating with the histone demethylase JMJ14. Nucleic Acids Res..

[28] Yang H., Han Z., Cao Y., Fan D., Li H., Mo H., Feng Y., Liu L., Wang Z., Yue Y. (2012). A companion cell-dominant and developmentally regulated H3K4 demethylase controls flowering time in Arabidopsis via the repression of FLC expression. PLoS Genet..

[29] Yang H., Mo H., Fan D., Cao Y., Cui S., Ma L. (2012). Overexpression of a histone H3K4 demethylase, JMJ15, accelerates flowering time in Arabidopsis. Plant Cell Rep..

[30] Lu S.X., Knowles S.M., Webb C.J., Celaya R.B., Cha C., Siu J.P., Tobin E.M. (2011). The Jumonji C domain-containing protein JMJ30 regulates period length in the Arabidopsis circadian clock. Plant Physiol..

[31] Shen Y., Silva N.C.e., Audonnet L., Servet C., Wei W., Zhou D.-X. (2014). Over-expression of histone H3K4 demethylase gene JMJ15 enhances salt tolerance in Arabidopsis. Front. Plant Sci..

[32] Huang S., Zhang A., Jin J.B., Zhao B., Wang T.J., Wu Y., Wang S., Liu Y., Wang J., Guo P. (2019). Arabidopsis histone H3K4 demethylase JMJ17 functions in dehydration stress response. New Phytol..

[33] Shen Y., Wu X., Liu D., Song S., Liu D., Wang H. (2016). Cold-dependent alternative splicing of a Jumonji C domain-containing gene MtJMJC5 in Medicago truncatula. Biochem. Biophys. Res. Commun..

[34] Liu G., Khan N., Ma X., Hou X. (2019). Identification, Evolution, and Expression Profiling of Histone Lysine Methylation Moderators in Brassica rapa. Plants.

[35] Qian Y., Chen C., Jiang L., Zhang J., Ren Q. (2019). Genome-wide identification, classification and expression analysis of the JmjC domain-containing histone demethylase gene family in maize. BMC Genom..

[36] Hou Y., Wang L., Wang L., Liu L., Li L., Sun L., Rao Q., Zhang J., Huang S. (2015). JMJ704 positively regulates rice defense response against Xanthomonas oryzae pv. oryzae infection via reducing H3K4me2/3 associated with negative disease resistance regulators. BMC Plant Biol..

[37] Li T., Chen X., Zhong X., Zhao Y., Liu X., Zhou S., Cheng S., Zhou D.-X. (2013). Jumonji C domain protein JMJ705-mediated removal of histone H3 lysine 27 trimethylation is involved in defense-related gene activation in rice. Plant Cell.

[38] Nardeli S.M., Artico S., Aoyagi G.M., de Moura S.M., da Franca Silva T., Grossi-de-Sa M.F., Romanel E., Alves-Ferreira M. (2018). Genome-wide analysis of the MADS-box gene family in polyploid cotton (Gossypium hirsutum) and in its diploid parental species (Gossypium arboreum and Gossypium raimondii). Plant Physiol. Biochem..

[39] Li F., Fan G., Lu C., Xiao G., Zou C., Kohel R.J., Ma Z., Shang H., Ma X., Wu J. (2015). Genome sequence of cultivated Upland cotton (Gossypium hirsutum TM-1) provides insights into genome evolution. Nat. Biotechnol..

[40] Wang K., Wang Z., Li F., Ye W., Wang J., Song G., Yue Z., Cong L., Shang H., Zhu S. (2012). The draft genome of a diploid cotton Gossypium raimondii. Nat. Genet..

[41] Zhang T., Hu Y., Jiang W., Fang L., Guan X., Chen J., Zhang J., Saski C.A., Scheffler B.E., Stelly D.M. (2015). Sequencing of allotetraploid cotton (Gossypium hirsutum L. acc. TM-1) provides a resource for fiber improvement. Nat. Biotechnol..

[42] Hu Y., Chen J., Fang L., Zhang Z., Ma W., Niu Y., Ju L., Deng J., Zhao T., Lian J. (2019). Gossypium barbadense and Gossypium hirsutum genomes provide insights into the origin and evolution of allotetraploid cotton. Nat. Genet..

[43] Paterson A.H., Wendel J.F., Gundlach H., Guo H., Jenkins J., Jin D., Llewellyn D., Showmaker K.C., Shu S., Udall J. (2012). Repeated polyploidization of Gossypium genomes and the evolution of spinnable cotton fibres. Nature.

[44] Ashraf J., Zuo D., Wang Q., Malik W., Zhang Y., Abid M.A., Cheng H., Yang Q., Song G. (2018). Recent insights into cotton functional genomics: Progress and future perspectives. Plant Biotechnol. J..

[45] Chen Z.J., Scheffler B.E., Dennis E., Triplett B.A., Zhang T., Guo W., Chen X., Stelly D.M., Rabinowicz P.D., Town C.D. (2007). Toward sequencing cotton (Gossypium) genomes. Plant Physiol..

[46] Zhu Y.X., Li F.G. (2013). The Gossypium raimondii genome, a huge leap forward in cotton genomics. J. Integr. Plant Biol..

[47] Sunilkumar G., Campbell L.M., Puckhaber L., Stipanovic R.D., Rathore K.S. (2006). Engineering cottonseed for use in human nutrition by tissue-specific reduction of toxic gossypol. Proc. Natl. Acad. Sci. USA..

[48] Xu B., Gou J.Y., Li F.G., Shangguan X.X., Zhao B., Yang C.Q., Wang L.J., Yuan S., Liu C.J., Chen X.Y. (2013). A cotton BURP domain protein interacts with α-expansin and their co-expression promotes plant growth and fruit production. Mol. Plant.

[49] Gelfman S., Cohen N., Yearim A., Ast G. (2013). DNA-methylation effect on cotranscriptional splicing is dependent on GC architecture of the exon-intron structure. Genome Res..

[50] Felix G., Duran J.D., Volko S., Boller T. (1999). Plants have a sensitive perception system for the most conserved domain of bacterial flagellin. Plant J..

[51] Cui X., Lu F., Qiu Q., Zhou B., Gu L., Zhang S., Kang Y., Cui X., Ma X., Yao Q. (2016). REF6 recognizes a specific DNA sequence to demethylate H3K27me3 and regulate organ boundary formation in Arabidopsis. Nat. Genet..

[52] Gregory S.L., Kortschak R.D., Kalionis B., Saint R. (1996). Characterization of the dead ringer gene identifies a novel, highly conserved family of sequence-specific DNA-binding proteins. Mol. Cell. Biol..

[53] Musselman C.A., Kutateladze T.G. (2009). PHD fingers epigenetic effectors and potential drug targets. Mol. Interv..

[54] Madsen B., Tarsounas M., Burchell J.M., Hall D., Poulsom R., Taylor-Papadimitriou J. (2003). PLU-1, a transcriptional repressor and putative testis-cancer antigen, has a specific expression and localisation pattern during meiosis. Chromosoma.

[55] Zhang S., Tian Z., Li H., Guo Y., Miao Y. (2019). Genome-wide analysis and characterization of F-box gene family in Gossypium hirsutum L.. BMC Genom..

[56] Hernandez-Garcia C.M., Finer J.J. (2014). Identification and validation of promoters and cis-acting regulatory elements. Plant Sci..

[57] Ku Y.S., Sintaha M., Cheung M.Y., Lam H.M. (2018). Plant hormone signaling crosstalks between biotic and abiotic stress responses. Int. J. Mol. Sci..

[58] Peleg Z., Blumwald E. (2011). Hormone balance and abiotic stress tolerance in crop plants. Curr. Opin. Plant Biol..

[59] Cao X., Costa L.M., Biderre-Petit C., Kbhaya B., Dey N., Perez P., McCarty D.R., Gutierrez-Marcos J.F., Becraft P.W. (2007). Abscisic acid and stress signals induce viviparous1 expression in seed and vegetative tissues of maize. Plant Physiol..

[60] Ross J.J., Reid J.B., Swain S.M., Hasan O., Poole A.T., Hedden P., Willis C.L. (1995). Genetic-regulation of gibberellin deactivation in pisum. Plant J..

[61] Xu Z., Sun M., Jiang X., Sun H., Dang X., Cong H., Qiao F. (2018). Glycinebetaine biosynthesis in response to osmotic stress depends on jasmonate signaling in watermelon suspension cells. Front. Plant Sci..

[62] Abe H., YamaguchiShinozaki K., Urao T., Iwasaki T., Hosokawa D., Shinozaki K. (1997). Role of Arabidopsis MYC and MYB homologs in drought- and abscisic acid-regulated gene expression. Plant Cell.

[63] Mirouze M., Paszkowski J. (2011). Epigenetic contribution to stress adaptation in plants. Curr. Opin. Plant Biol..

[64] Chen X., Hu Y., Zhou D.-X. (2011). Epigenetic gene regulation by plant Jumonji group of histone demethylase. Biochim. Biophys. Acta..

[65] Huang Y., Chen D., Liu C., Shen W., Ruan Y. (2016). Evolution and conservation of JmjC domain proteins in the green lineage. Mol. Genet. Genomics.

[66] Zhao W., Shafiq S., Berr A., Shen W.H. (2015). Genome-wide gene expression profiling to investigate molecular phenotypes of Arabidopsis mutants deprived in distinct histone methyltransferases and demethylases. Genom Data..

[67] Han Y., Li X., Cheng L., Liu Y., Wang H., Ke D., Yuan H., Zhang L., Wang L. (2016). Genome-wide analysis of soybean JmjC domain-containing proteins suggests evolutionary conservation following whole-genome duplication. Front. Plant Sci..

[68] Gu T., Han Y., Huang R., McAvoy R.J., Li Y. (2016). Identification and characterization of histone lysine methylation modifiers in Fragaria vesca. Sci. Rep..

[69] Dong Y., Lu J., Liu J., Jalal A., Wang C. (2020). Genome-wide identification and functional analysis of JmjC domain-containing genes in flower development of Rosa chinensis. Plant Mol. Biol..

[70] Zong W., Zhong X., You J., Xiong L. (2013). Genome-wide profiling of histone H3K4-tri-methylation and gene expression in rice under drought stress. Plant Mol. Biol..

[71] Wilsker D., Patsialou A., Dallas P.B., Moran E. (2002). ARID proteins: A diverse family of DNA binding proteins implicated in the control of cell growth, differentiation, and development. Cell Growth Differ..

[72] Kim H.J., Triplett B.A. (2001). Cotton fiber growth in planta and in vitro. Models for plant cell elongation and cell wall biogenesis. Plant Physiol..

[73] Wang X., Gao J., Gao S., Li Z., Kuai B., Ren G. (2019). REF6 promotes lateral root formation through de-repression of PIN1/3/7 genes. J. Integr. Plant Biol..

[74] Zhu T., Liang C., Meng Z., Sun G., Meng Z., Guo S., Zhang R. (2017). CottonFGD: An integrated functional genomics database for cotton. BMC Plant Biol..

[75] El-Gebali S., Mistry J., Bateman A., Eddy S.R., Luciani A., Potter S.C., Qureshi M., Richardson L.J., Salazar G.A., Smart A. (2019). The Pfam protein families database in 2019. Nucleic Acids Res..

[76] Letunic I., Copley R.R., Schmidt S., Ciccarelli F.D., Doerks T., Schultz J., Ponting C.P., Bork P. (2004). SMART 4.0: Towards genomic data integration. Nucleic Acids Res..

[77] Gasteiger E., Gattiker A., Hoogland C., Ivanyi I., Appel R.D., Bairoch A. (2003). ExPASy: The proteomics server for in-depth protein knowledge and analysis. Nucleic Acids Res..

[78] Li H., Guan H., Zhuo Q., Wang Z., Li S., Si J., Zhang B., Feng B., Kong L.A., Wang F. (2020). Genome-wide characterization of the abscisic acid-, stress- and ripening-induced (ASR) gene family in wheat (Triticum aestivum L.). Biol. Res..

[79] Larkin M.A., Blackshields G., Brown N.P., Chenna R., McGettigan P.A., McWilliam H., Valentin F., Wallace I.M., Wilm A., Lopez R. (2007). Clustal W and Clustal X version 2.0. Bioinformatics.

[80] Kumar S., Stecher G., Tamura K. (2016). MEGA7: Molecular evolutionary genetics analysis version 7.0 for bigger datasets. Mol. Biol. Evol..

[81] Liu W., Li W., He Q., Daud M.K., Chen J., Zhu S. (2015). Characterization of 19 genes encoding membrane-bound fatty acid desaturases and their expression profiles in Gossypium raimondii under low temperature. PLoS ONE.

[82] Li W., Sun K., Ren Z., Song C., Pei X., Liu Y., Wang Z., He K., Zhang F., Zhou X. (2018). Molecular evolution and stress and phytohormone responsiveness of SUT genes in Gossypium hirsutum. Front. Genet..

[83] Chen C., Chen H., Zhang Y., Thomas H.R., Frank M.H., He Y., Xia R. (2020). TBtools: An integrative toolkit developed for interactive analyses of big biological data. Mol. Plant.

[84] Hu B., Jin J., Guo A.Y., Zhang H., Luo J., Gao G. (2015). GSDS 2.0: An upgraded gene feature visualization server. Bioinformatics.

[85] Lescot M., Dehais P., Thijs G., Marchal K., Moreau Y., Van de Peer Y., Rouze P., Rombauts S. (2002). PlantCARE, a database of plant cis-acting regulatory elements and a portal to tools for in silico analysis of promoter sequences. Nucleic Acids Res..

