# Supplementary figures and images for: Characterization and Stress Response of the JmjC Domain-Containing Histone Demethylase Gene Family in the Allotetraploid Cotton Species Gossypium hirsutum
Source: Plants (Basel). 2020 Nov 20;9(11):1617. doi: 10.3390/plants9111617 (PMC7709011; doi:10.3390/plants9111617)

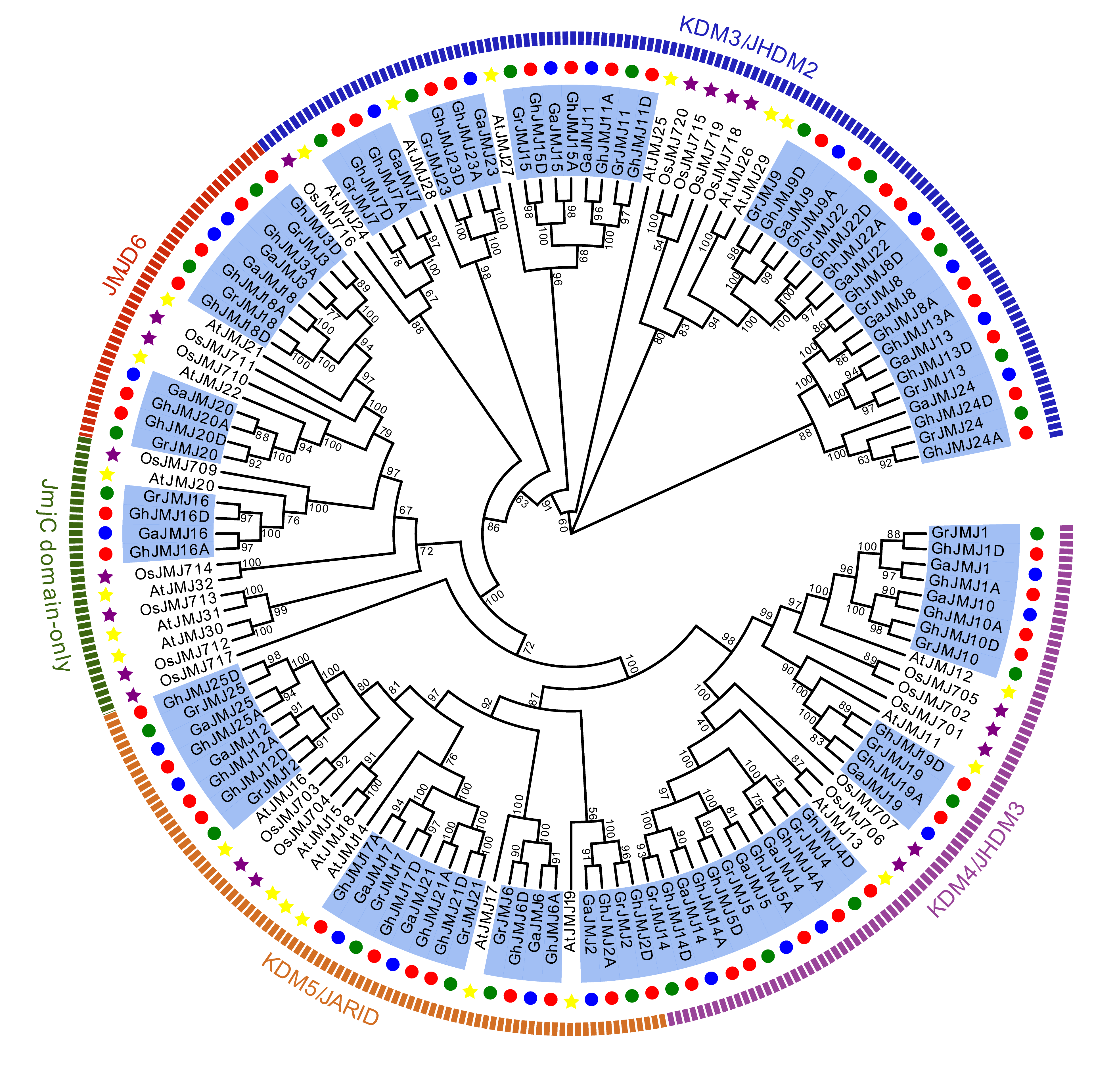

Supplement: Supplementary file 1 [file plants-09-01617-s001.zip › Supplementary Files/Figure S1.tif]

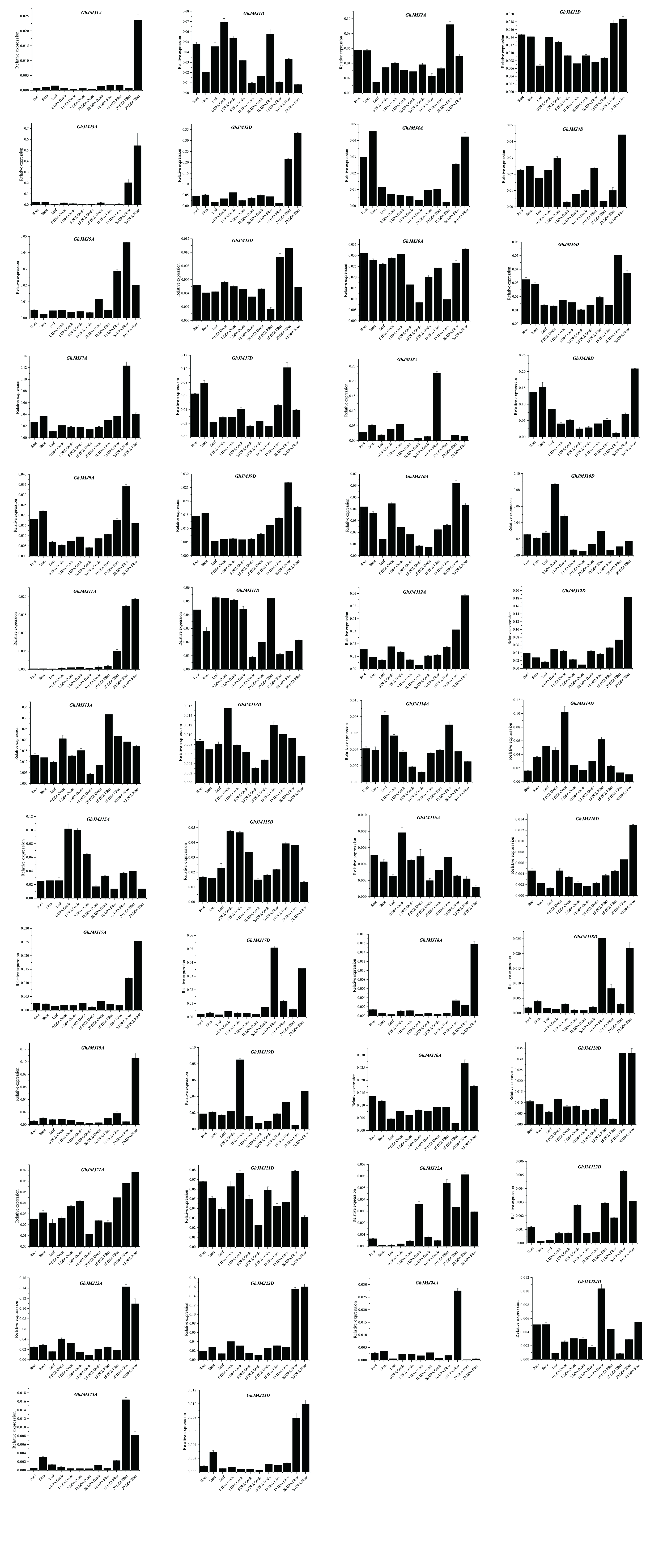

Supplement: Supplementary file 1 [file plants-09-01617-s001.zip › Supplementary Files/Figure S3.tif]

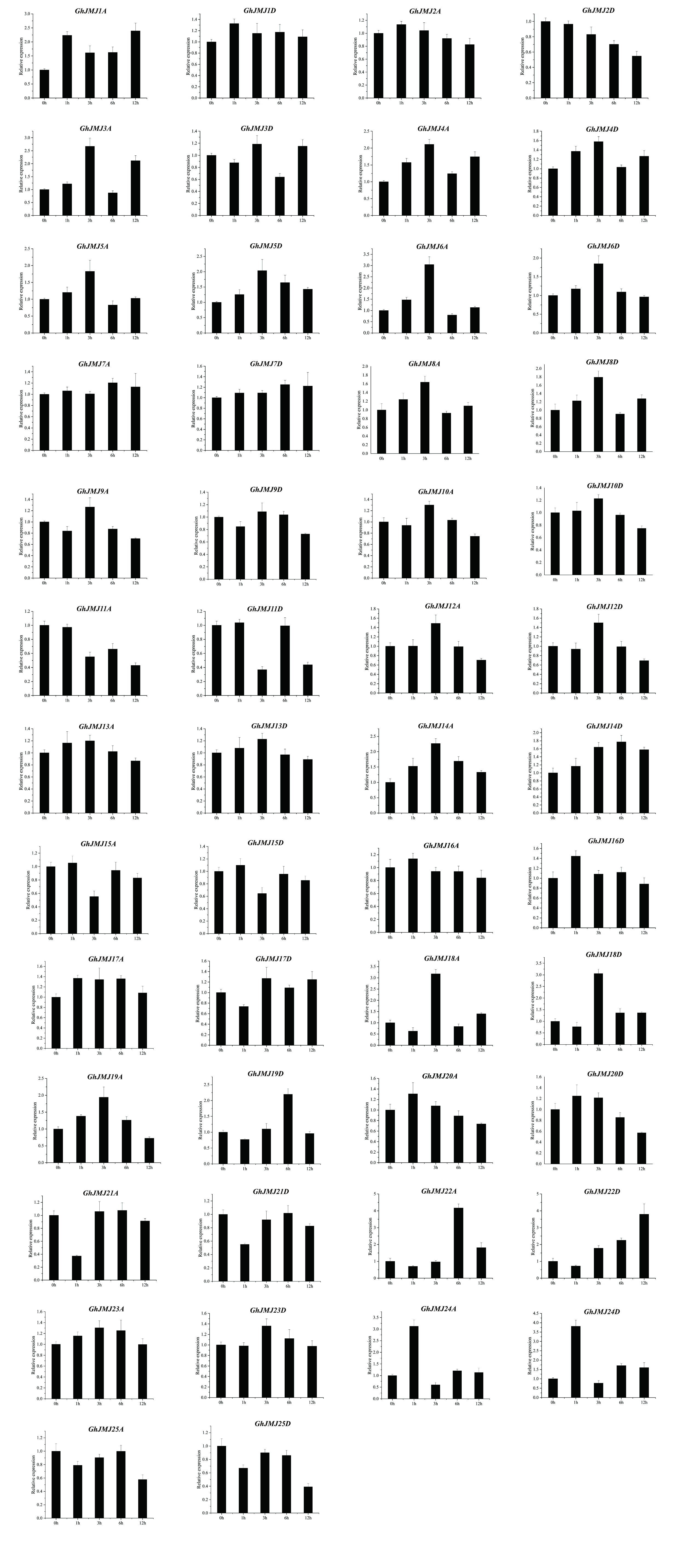

Supplement: Supplementary file 1 [file plants-09-01617-s001.zip › Supplementary Files/Figure S4.tif]

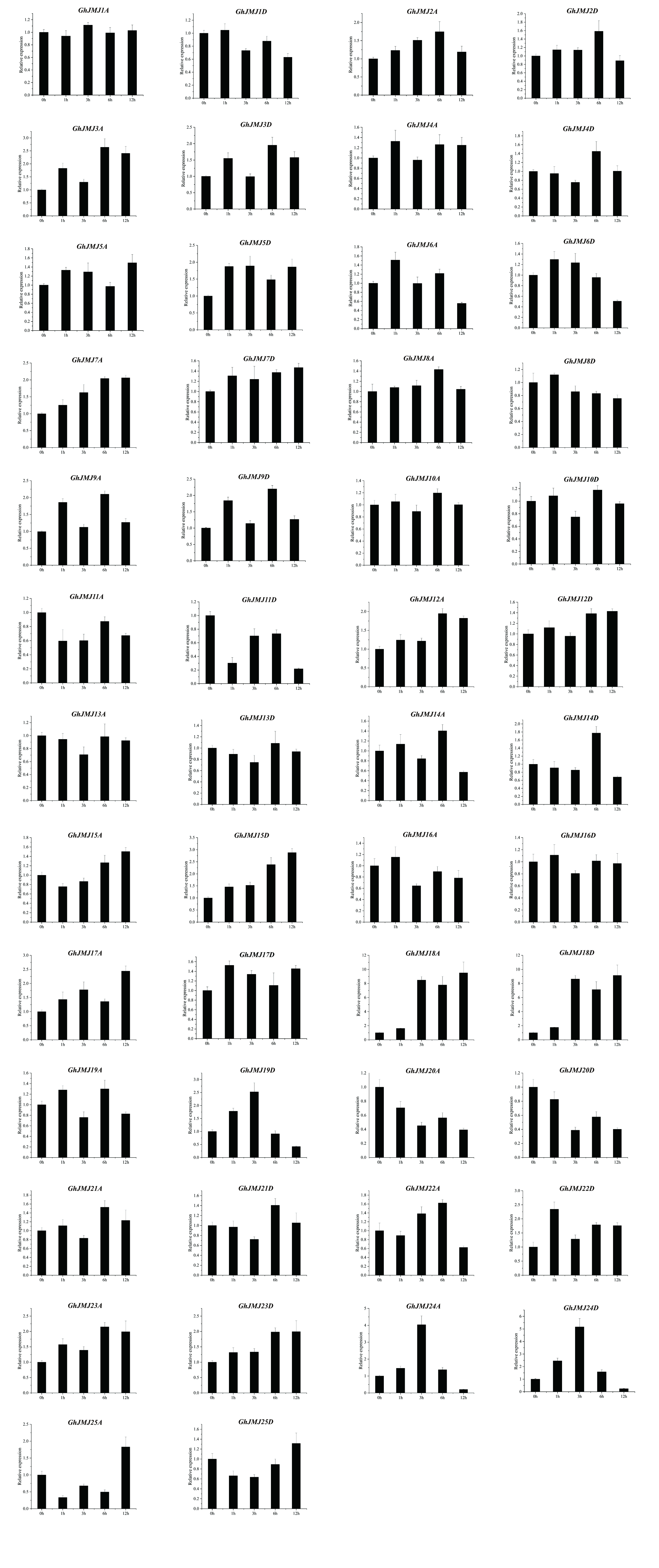

Supplement: Supplementary file 1 [file plants-09-01617-s001.zip › Supplementary Files/Figure S5.tif]
